# Establishment and application of multiplex PCR method for detection of *Trichophyton verrucosum*, *Microsporum canis*, and *Trichophyton mentagrophytes* from cattle

**DOI:** 10.3389/fvets.2025.1546586

**Published:** 2025-03-24

**Authors:** Ya Wang, Zhiguo Li, Zhicai Zuo, Xiaobin Gu, Dongjie Cai, Jianfeng Hu, Yu Gu, Liuhong Shen, Liping Gou, Kun Zhang, Xiaoping Ma

**Affiliations:** ^1^College of Veterinary Medicine, Sichuan Agricultural University, Chengdu, China; ^2^Key Laboratory of Animal Disease and Human Health of Sichuan Province, Chengdu, China; ^3^Institute of New Rural Development, Sichuan Agricultural University, Chengdu, China; ^4^College of Life Science, Sichuan Agricultural University, Chengdu, China

**Keywords:** dermatophytosis, *Trichophyton verrucosum*, *Microsporum canis*, *Trichophyton mentagrophytes*, multiplex PCR

## Abstract

**Introduction:**

Dermatophytosis, which is a contagious fungal skin infection common in animals and humans, is the most common skin disease in cattle. It has a serious negative impact on the livestock industry. In order to circumvent the shortcomings of traditional detection methods such as time-consuming and low isolation rate. Therefore, this study established a simple, rapid and effective diagnostic method to accurately diagnose and differentiate the causative fungi of dermatophytosis, which is of great significance to enhance the prevention and treatment of dermatophytosis in beef cattle farms.

**Methods:**

Three pairs of specific primers were designed using Primer Premier 5.0 from *Trichophyton verrucosum*, *Microsporum canis* and *Trichophyton mentagrophytes*. A triple PCR assay was established by optimising the primer dose and annealing temperature to improve the detection sensitivity. The feasibility of the method was verified by testing the samples.

**Results and discussion:**

In this study, a multiplex PCR method that can rapidly detect these three fungi at the same time was established, and its specificity, sensitivity and repeatability were analyzed at the same time. The results showed that the multiplex PCR method amplified the specific expected fragments of 581 bp, 1,513 bp and 371 bp for *T. verrucosum*, *M. canis* and *T. mentagrophytes*. The minimum detection limits of *T. verrucosum, M. canis* and *T. mentagrophytes* were all 1 pg./μL. The positive rates were 87.5% (21/24) for samples. The results showed that the multiplex PCR method was simple, specific and sensitive and might be used for rapid diagnosis and identification of dermatophytes in cattle.

## 1 Introduction

Dermatophytosis is a fungal infection caused by dermatophytes (1) that is highly contagious and affects both animals and humans (2, 3). It is mainly caused by a group of fungi in the genera *Trichophyton*, *Microsporum* and *Epidermophyton* (4). And it is a significant concern in global public health and veterinary practice (5). The consequences of this phenomenon for cattle farmers are manifold. Among the most significant are the following: reduced weight gain, diminished milk production, deferred marketing and a decline in the quality of raw hide materials (3, 6).

Most bovine dermatophytosis caused by *T. verrucosum*, although *T. mentagrophytes* and *M. canis* can also cause dermatophytosis in cattle (7). The traditional methods of detecting dermatophytes, such as microscopic examination and fungal culture, have multiple drawbacks. They are time-consuming and labor-intensive, requiring highly skilled diagnosticians as similar species share morphological similarities, limiting discriminatory power (8, 9). Furthermore, accurate diagnosis through traditional isolation cultures is only possible after apparent clinical symptoms develop, potentially leading to missed treatment opportunities or inappropriate medication administration (10). So, rapid and convenient detection methods are important in practice. PCR-based molecular tests have emerged, but many, like real-time PCR (11), nested PCR (12), etc., are complex to operate and have high equipment requirements. Multiplex PCR offers several advantages. It simultaneously incorporates multiple pairs of primers into a reaction system and amplifies numerous target fragments with the utilization of multiple DNA templates (13). And its ease of operation and low cost make it accessible, especially for farms that may not have advanced laboratory facilities. It has low equipment requirements, which reduces the time and cost associated with sending samples to specialized labs. It can detect pathogens even in the latent phase of the disease (14), which is crucial as early detection can prevent further spread and economic losses. In recent times, by virtue of its characteristics of quicker speed, greater sensitivity, and more pronounced specificity, multiplex PCR has been widely employed (15–17).

In addition, the proportion of drug-resistant strains increases significantly over time (18–20), and drug susceptibility may also vary between strains (21–23), which may lead to the need for different dosing regimens during treatment. Therefore, targeted treatment is essential for disease control and elimination. To address this need, establishing an assay technology capable of rapid and efficient diagnosis is critical.

Currently, several PCR-based assays exist for the detection of *T. verrucosum* (24), *T. mentagrophytes* (25), and *M. canis* (26), but only one type of fungus can be detected. Although a multiplex PCR assay has been developed for the simultaneous detection of *T. mentagrophytes* and *M. canis* in canine dermatophytosis (17), a similar comprehensive method for cattle is lacking. This study establishes a simple, rapid and effective diagnostic method to accurately detect and differentiate dermatophyte species affecting beef cattle farms. By implementing this approach, preventive and control strategies can be formulated to mitigate adverse impacts on cattle health and production performance, reduce economic losses, and strengthen dermatophyte management practices in beef cattle farming systems.

## 2 Materials and methods

### 2.1 Fungal strain and cultivation

*T. verucosum*, *M. canis* and *T. mentagrophytes* were used as target strains for multiplex PCR detection. The specificity of the primers was verified by using four strains of *M. gypseum*, *T. rubrum*, *Trichosporon asahii* and *Trichosporon loubieri* as negative controls. The strains are preserved at the College of Veterinary Medicine, Sichuan Agricultural University. The strains were resuscitated by inoculating them into Sabouraud Dextrose Agar medium (Sangon Biotech, Beijing, China) for 5–7 days at 27°C.

### 2.2 DNA extraction of pathogens and clinical samples

The rapid Fungi Genomic DNA Isolation Kit (27) (Sangon Biotech, Shanghai, China) was used to extract genomic DNA, following the manufacturer’s instructions. The specific steps are as follows: Take 50—100 mg of fungal hyphae and clinical samples, grind them to a powder using liquid nitrogen and place in a 1.5 mL centrifuge tube. Next, 400 μL of Buffer Digestion and 4 μLof 2-Mercaptoethanol MCH were added and mixed well with shaking. The tube is then placed in a water bath at 65°C for 1 h until the sample is completely cleaved. At the end of the water bath, RNA was removed by adding 20 μL of RNaseA at a concentration of 10 mg/mL and left at room temperature for 2–5 min. Add 200 μL of Buffer PF, mix thoroughly by inversion, and leave at −20°C for 5 min. Centrifuge at 10,000 rpm for 5 min at room temperature, and transfer the supernatant (500 ~ 550 μL) into a new 1.5 mL centrifuge tube. Add an equal volume of isopropanol and mix well by inverting 5 ~ 8 times, and leave at room temperature for 2 ~ 3 min. Centrifuge at 10,000 rpm for 5 min at room temperature and discard the supernatant. Add 1 mL of 75% ethanol, rinse for 1 ~ 3 min, centrifuge at 10,000 rpm for 2 min and discard the supernatant (repeat the procedure once). Uncover and invert at room temperature for 5–10 min until the residual ethanol is completely evaporated. The obtained DNA was bath lysed with 50–100 μL TE Buffer. The concentration of DNA was determined by Ultra-micro spectrophotometer. The extracted DNA can be immediately used for the next step of the experiment or stored at −20°C.

### 2.3 Primer design

Specific primers were designed using Primer 5 software based on the asparaginase family protein gene (Gene ID: 9584630) of *T. verucosum*, the C6 zinc finger domain-containing protein gene (Gene ID: 9229418) of *M. canis*, and the mitochondrion (Sequence ID: NC_012826.1) of *T. mentagrophytes*. As shown in Supplementary Table S1, all primers were synthesized by Biochemical and Biological Engineering (Shanghai) Co., Ltd.

### 2.4 PCR amplification

The single PCR system is shown in Supplementary Table S2. Single PCR conditions: pre-denaturation at 94°C for 10 min, denaturation at 94°C for 30 s, annealing at 55°C for 30 s, extension at 72°C for 1 min 10s, followed by 30 cycles, and final extension at 72°C for 10 min. The PCR amplification products were electrophoresed with 1.2% agarose gel containing Goldview stain at 120 V for 20 min to detect the presence of target bands (28). Similar conditions were used for multiplex PCR, except for the annealing temperature.

### 2.5 Specificity of primers

The specificity of primers was assessed by amplifying different fungal DNA, including DNA from three targeted dermatophyte species (*T. verucosum*, *M. canis* and *T. mentagrophytes*), as well as negative controls (*M. gypseum*, *T. rubrum*, *T. asahii*, *T. loubieri* and ddH_2_0).

### 2.6 Optimization of multiplex PCR

The optimization of important factors affecting multiplex PCR amplification was carried out by adding the genomic DNA and specific primers of three targeted dermatophyte species to the conventional PCR reaction simultaneously. The primer volumes were added to different reaction systems according to the amounts specified in Supplementary Table S3 (29). The annealing temperature was set to 50°C, 52°C, 54°C, 55°C, 56°C, 57°C, 58°C, 59°C, 60°C, 62°C.

### 2.7 Sensitivity analysis of single and multiplex PCR

First, dilute the initial template concentration of DNA of *T. verucosum*, *M. canis* and *T. mentagrophytes* to 10 ng/μL and then serially dilute tenfold (10 ng to 1 pg) with TE Buffer (Sangon Biotech, Shanghai, China). Single and multiplex PCR were performed under optimized conditions (30).

### 2.8 Reproducibility of multiplex PCR

The reproducibility test was carried out on the mixed DNA template of *T. verrucosum*, *T. mentagrophytes* and *M. canis* by using the optimized multiplex PCR reaction system, and the test was repeated 10 times.

### 2.9 Collection of clinical samples and fungal cultures

We collected 24 clinical scales and hair samples from cattle suspected of having dermatophytosis in Sichuan Province, China. After the clinical samples were examined microscopically, the samples suspected to be infected with dermatophyte were inoculated in SDA medium using the three-point inoculation method and incubated for 7–14 d at 27°C. The growth was observed daily, and a single colony was selected for further purification to obtain a stable colony (31, 32). The fungi cultures were identified on their morphology characteristics and PCR amplification and sequencing of internal tran (ITS) (33–35).

### 2.10 Multiplex PCR detection of pathogenic fungi in clinical samples

Total DNA was extracted from the collected clinical samples using an established multiplex PCR system and tested.

## 3 Results

### 3.1 Multiplex PCR primer specificity amplification

**Figure 1:**
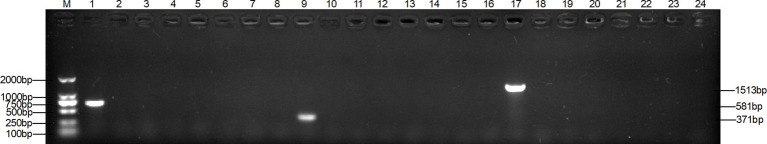
Specificity of single PCR. Lanes 1–8: *T. verrucosum*, *T. mentagrophytes*, *M. canis*, *T. loubieri*, *M. gypsum*, *T. rubrum* and ddH_2_0; lanes 9–16: *T. mentagrophytes*, *T. verrucosum*, *M. canis*, *T. loubieri*, *M. gypsum*, *T. rubrum* and ddH_2_0.; lanes 17–24: *M. canis*, *T. verrucosum*, *T. mentagrophytes*, *T. loubieri*, *M. gypsum*, *T. rubrum* and ddH_2_0. The DNA template of each pathogen was amplified using primers TAFP-1/TAFP-4, QM-1/QM-4, and XM-1/XM-4 in lanes 1–8, 9–16, and 17–24, respectively. ddH_2_0 is nuclease-free and sterile water; the band sizes of *T. verrucosum*, *T. mentagrophytes*, and *M. canis* were 581 bp, 371 bp, and 1,513 bp, respectively, and the following figures all represent these.

**Figure 2:**
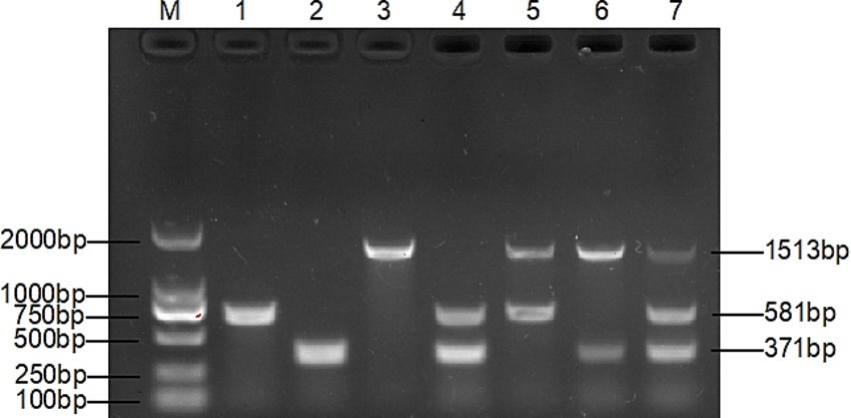
Specificity of multiplex PCR. Lane 1: *T. verrucosum*, lane 2: *T. mentagrophytes*, lane 3: *M. canis*, lane 4: *T. verrucosum* and *T. mentagrophytes*, lane 5: *T. verrucosum* and *M. canis*, lane 6: *M. canis* and *T. mentagrophytes*, lane 7: *T. verrucosum*, *T. mentagrophytes* and *M. canis*, lane 1–8 all have 3 pairs of primers added, indicating that the three pairs of primers do not interact with each other.

The multiplex PCR assay yielded fragments of the expected size using *T. verucosum*, *M. canis* and *T. mentagrophytes* as genomic DNA with specific primers (Figure 1), while no products were obtained with non-target species (Figure 2), thereby validating the specificity of the multiplex PCR assay.

### 3.2 Optimization of multiplex PCR

**Figure 3:**
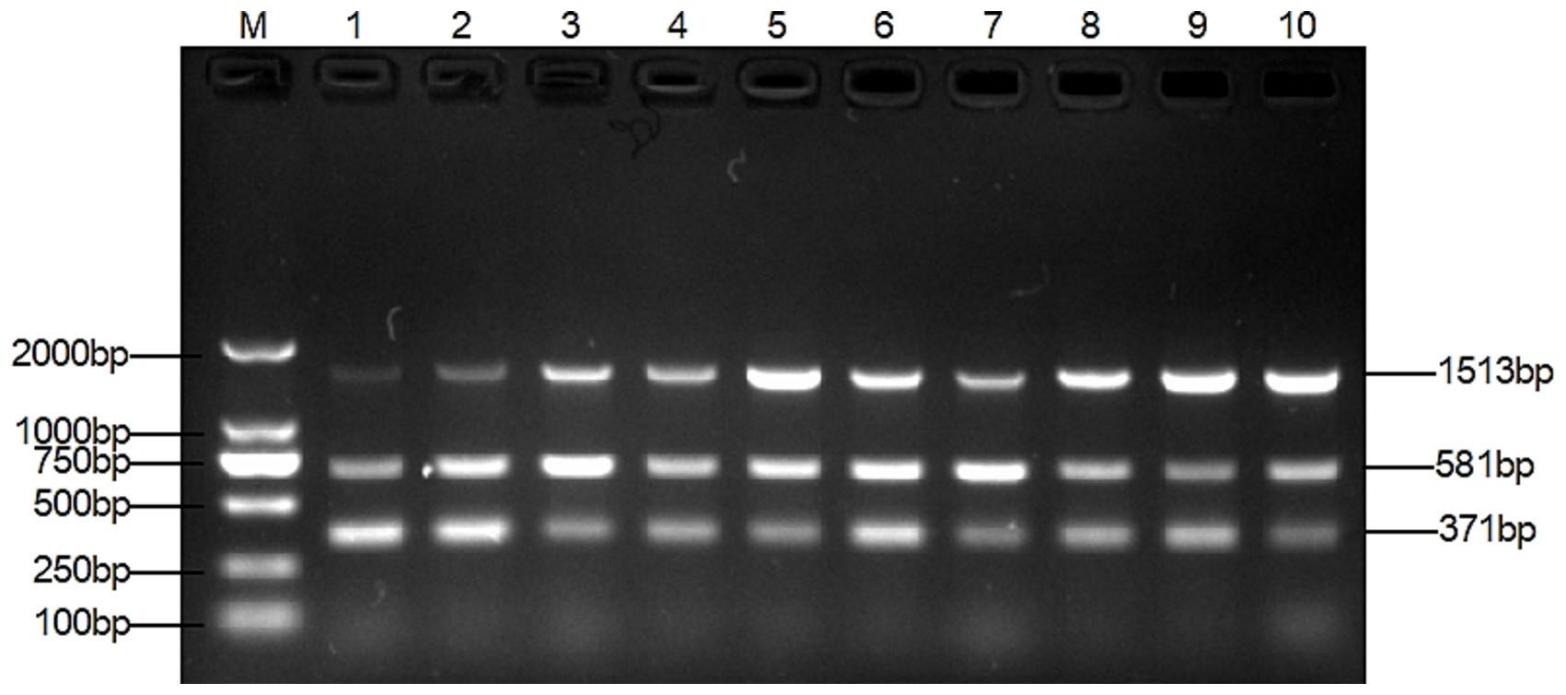
Optimization of the optimal primer doses for multiplex PCR. Lane M: DL2000 marker, lanes 1–10: primer doses were added according to Supplementary Table S3.

**Figure 4:**
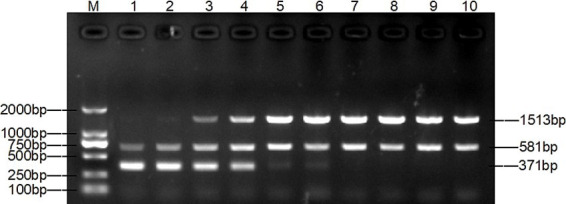
Optimization of the annealing temperature for multiplex PCR. Lane M: DL2000 marker, lanes 1–10: 50°C, 52°C, 54°C, 55°C, 56°C, 57°C, 58°C, 59°C, 60°C, 62°C.

The optimal primer concentration was achieved when 0.4 μL of TAFP-1/TAFP-4, 1 μL of QC6-1/QC6-4, and 0.4 μL of XM-1/XM-1 were added, resulting in three distinct bands (Figure 3). Additionally, Figure 4 shows that three clear strips were obtained when the annealing temperature was increased from 50°C to 57°C, with the strips clearest at 55°C. However, when the annealing temperature was ≥58°C, multiplex PCR failed to amplify the target bands of *Trichophyton* species.

### 3.3 Sensitivity of the single and multiplex PCR

**Figure 5:**
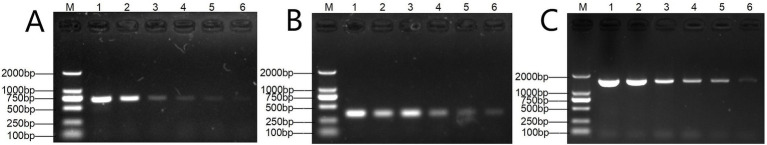
Sensitivity of the single PCR assay for *T. verucosum* **(A)**, *T. mentagrophytes* **(B)** and *M. canis* **(C)**. Lane M: DL2000 marker, lanes 1–7: DNA concentrations were 10 ng/μL, 1 ng/μL, 500 pg./μL, 100 pg./μL, 10 pg./μL, and 1 pg./μL, respectively.

**Figure 6:**
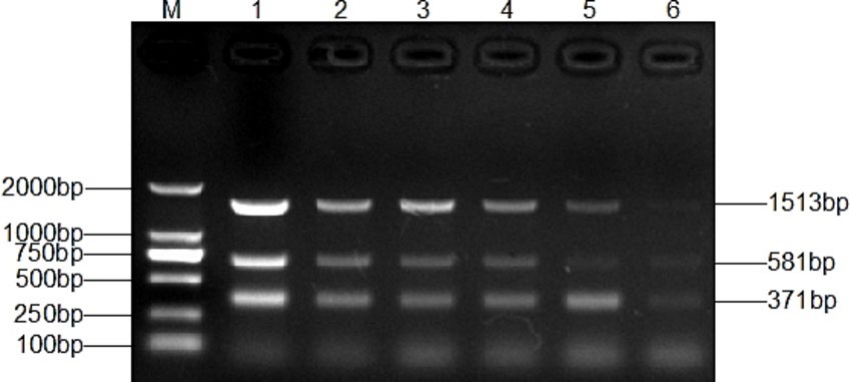
Sensitivity of the multiplex PCR assay for *T. verucosum*, *T. mentagrophytes* and *M. canis*. Lane M: DL2000 marker, Lanes 1-7: DNA concentrations were 10 ng/μL, 1 ng/μL, 500 pg/μL, 100 pg/μL, 10 pg/μL, and 1 pg/μL, respectively.

Genomic DNA was sequentially diluted for the sensitivity assay of single and multiplex PCR. A single PCR amplification sensitivity test detected 1 pg. DNA for *T. verucosum*, *M. canis* and *T. mentagrophytes*, respectively (Figure 5), while the multiplex PCR detected 1 pg. for *T. verucosum*, *M. canis* and *T. mentagrophytes*, respectively (Figure 6).

### 3.4 Reproducibility of multiplex PCR

The above specific detection was repeated 10 times and the results remained unchanged (Supplementary Figure S1).

### 3.5 Isolation of fungi

We isolated 5 strains of *T. verrucosum* (GenBank: OR056436.1, PQ495702.1, PQ495703.1, PQ495704.1, and PQ495705.1) from 4 of 24 clinical samples and did not isolate *T. mentagrophytes* and *M. canis*.

### 3.6 Application of multiplex PCR assays in clinical samples

DNA extraction and multiplex PCR analysis were performed on all 24 collected samples. As shown in Supplementary Figure S2, the overall detection positivity rate reached 87.5% (21/24), with the following species-specific results: *T. verrucosum* was identified in 16 samples (1 inconclusive), *T. mentagrophytes* in 7 samples (2 inconclusive), and *M. canis* in 10 samples (all test bands ambiguous). Notably, mixed infections were observed, featuring concurrent *T. verrucosum* and *T. mentagrophytes* infections, or combined *T. verrucosum* and *M. canis* infections.

## 4 Discussion

Dermatophytosis is prevalent among cattle and ranks as one of the most common skin ailments. These conditions often persist over extended periods, involve concurrent fungal infections, and pose challenges in terms of isolation and cultivation. Presently, the main methods employed in diagnosing dermatomycosis clinically encompass Wood’s lamp illumination, direct microscopic examination, and fungal culture (36–38). These detection methods exhibit specific constraints. For instance, consider fungal isolation and culture: it involves labor-intensive procedures, lengthy durations, and results in a low isolation rate (39). Hence, the establishment of a simple, rapid and effective diagnostic method that can precisely diagnose and distinguish the pathogenic fungi of dermatophytosis causing dermatophytosis is crucial for enhancing the prevention and treatment of this condition in beef cattle farms.

In this study, dermatophytosis cultures were isolated in only 16.67% of samples (4/24), and the methodology relied on subjective evaluation, potentially leading to false-positive or false-negative results (24). Animal dermatophytosis is typically the result of a mixed fungal infection involving one or more fungi (40). Multiplex PCR technology has emerged as a widely utilized approach for the clinical identification of pathogens. Multiplex PCR utilizes multiple pairs of primers to amplify multiple nucleic acid bands simultaneously, enabling the simultaneous identification and differentiation of mixed fungi (41). In reality, the screening and identification of dermatomycosis play a crucial role in guiding significance.

After many pre-experiments, we finally chose three genes that are highly conserved and stable, and can provide a precise target for designing specific primers. On the basis of this, three pairs of new primers with high specificity and sensitivity were successfully designed, and a multiplex PCR assay was established accordingly for the detection of the three dermatophytes. The three pairs of specific primers did not interfere with each other (Figure 2), and there was no amplification for other control strains (Figure 1).

The method was optimised, evaluated, and applied clinically. The key to the success of multiplex PCR is to optimise the concentration of primers and the annealing temperature, so that each set of primers can effectively amplify its target DNA without interference from other primers. The test results were in agreement with previous studies that both primer volumes and annealing temperature had an effect on the test results (29, 35). The dose of primers can affect the amplification efficiency of multiplex PCR. As shown in lane 1 of Figure 3, the target band of *M. canis* is darker when the primer concentration is the same. By gradually reducing the dose of primers for *T. mentagrophytes* and *T. verrucosum*, the target band of *M. canis* gradually becomes clearer. The annealing temperature also affects the amplification efficiency of the multiplex PCR. As shown in lanes 1 and 2 of Figure 3, *M. canis* does not have a target band when the annealing temperature is the same. Various temperature levels are used to modify the annealing temperature. As shown in lane 4 of Figure 4, the target bands of *T. verrucosum*, *T. mentagrophytes* and *M. canis* are most prominent at 55°C.

Sensitivity is also a key part of making multiplex PCR more efficient. The multiplex PCR method constructed in this study showed high sensitivity in the detection of *M. canis* and *T. mentagrophytes*. Specifically, the lowest detection concentration of this method for *M. canis* and *T. mentagrophytes* was 1 pg./μL, which contrasted with that of 12.6 pg./μL for *M. canis* and 13.6 pg./μL for T*. mentagrophytes* in the triple PCR method constructed by Qian et al. (17), and highlighted the significant advantages of the method in terms of the lowest detection concentration (Figure 6). Compared to single PCR assays, this multiplex PCR allows for the simultaneous amplification of specific bands for all three fungi (*T. verrucosum*, *M. canis*, and *T. mentagrophytes*) with similar detection sensitivity (Figure 5), thus improving the clinical detection rate. Furthermore, unlike PCR methods from other studies (42–44), our method only requires DNA extraction from the sample to detect pathogenic fungal infections, eliminating the need for pure cultures, which makes the assay more efficient and convenient.

As shown in Supplementary Figure S2, the positive rate of the sample was 87.5% (21/24). From the results of our study, it is clear that *T. verrucosum* is the main cause of dermatophytic infections in cattle, which is in accordance with the reports of previous studies (12). Although *T. verrucosum* is the main cause of infection, *T. mentagrophytes* and *M. canis* can also cause infections in cattle, which is consistent with the reports of Dalis et al. (5) and Abu-Samra et al. (45). The results also demonstrate the presence of mixed fungal infections in dermatophytosis, supporting the feasibility of our diagnostic approach.

## Data Availability

The original contributions presented in the study are included in the article/Supplementary material, further inquiries can be directed to the corresponding author.

## Supplementary material

SUPPLEMENTARY FIGURE S1 Repeatability of the multiplex PCR assay. 1-10 indicates the results of 10 repetitions of the constructed multiplex PCR method on different days or by different experimenters.

SUPPLEMENTARY FIGURE S2 Multiplex PCR methods applied to clinical samples detection in some areas of Sichuan Province. **(A)** Represents samples from 1-10 and **(B)** represents samples from 11-24. Lane M: DL2000 marker; Lane 1-24: Clinical sample detection. If a 581 bp band appears in the detection results, it indicates that the sample contains *T. verrucosum*. If a 1513 bp band appears, it suggests the presence of *M. canis*. If a 371 bp band appears, it means the sample contains *T. mentagrophytes*. In the absence of any bands, it implies that the infection of this sample is not caused by the above three fungi.
