# Supplementary figures and images for: Establishment and application of multiplex PCR method for detection of Trichophyton verrucosum, Microsporum canis, and Trichophyton mentagrophytes from cattle
Source: Front Vet Sci. 2025 Mar 24;12:1546586. doi: 10.3389/fvets.2025.1546586 (PMC11973286; doi:10.3389/fvets.2025.1546586)

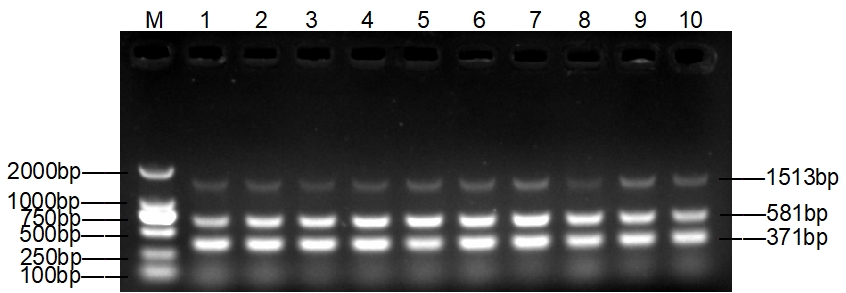

Supplement: SUPPLEMENTARY FIGURE S1 — Repeatability of the multiplex PCR assay. 1-10 indicates the results of 10 repetitions of the constructed multiplex PCR method on different days or by different experimenters. [file Image_1.JPEG]

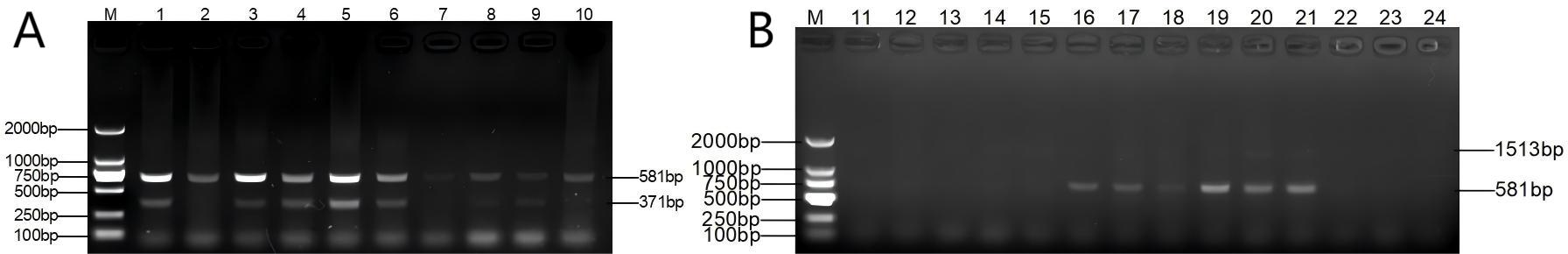

Supplement: SUPPLEMENTARY FIGURE S2 — Multiplex PCR methods applied to clinical samples detection in some areas of Sichuan Province. (A) Represents samples from 1-10 and (B) represents samples from 11-24. Lane M: DL2000 marker; Lane 1-24: Clinical sample detection. If a 581 bp band appears in the detection results, it indicates that the sample contains T. verrucosum. If a 1513 bp band appears, it suggests the presence of M. canis. If a 371 bp band appears, it means the sample contains T. mentagrophytes. In the absence of any bands, it implies that the infection of this sample is not caused by the above three fungi. [file Image_2.JPEG]
